# Sidewinder‐Inspired Self‐Adjusting, Lateral‐Rolling Soft Robots for Autonomous Terrain Exploration

**DOI:** 10.1002/advs.202308350

**Published:** 2024-01-29

**Authors:** Young Been Kim, Shu Yang, Dae Seok Kim

**Affiliations:** ^1^ Department of Polymer Engineering Pukyong National University 45 Yongso‐ro, Nam‐gu Busan 48513 South Korea; ^2^ Department of Materials Science and Engineering University of Pennsylvania 3231 Walnut Street Philadelphia PA 19104 USA

**Keywords:** helical filament, liquid crystal elastomer, self‐regulation, soft robotics

## Abstract

Helical structures of liquid crystal elastomers (LCEs) hold promise in soft robotics for self‐regulated rolling motions. The understanding of their motion paths and potentials for terrain exploration remains limited. This study introduces a self‐adjusting, lateral‐rolling soft robot inspired by sidewinder snakes. The spring‐like LCE helical filaments (HFs) autonomously respond to thermal cues, demonstrating dynamic and sustainable locomotion with adaptive rolling along non‐linear paths. By fine‐tuning the diameter, pitch, and modulus of the LCE HFs, and the environmental temperature, the movements of the LCE HFs, allowing for exploration of diverse terrains over a 600 cm^2^ area within a few minutes, can be programmed. LCE HFs are showcased to navigate through over nine obstacles, including maze escaping, terrain exploration, target hunting, and successfully surmounting staircases through adaptable rolling.

## Graphical abstract

Inspired by sidewinder snakes, liquid crystal elastomer helical filaments (LCE HFs) are prepared, demonstrating temperature‐activated self‐adjusts their movements. It can dynamically roll in non‐linear paths, responding to filament properties and environmental temperature. The robot showcases terrain adaptability, navigating through a 600 cm^2^ area with nine obstacles, including mazes, terrain exploration, target finding, and climbing stairs through self‐rolling.

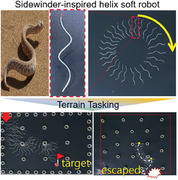

## 1 Introduction

**Figure 1:**
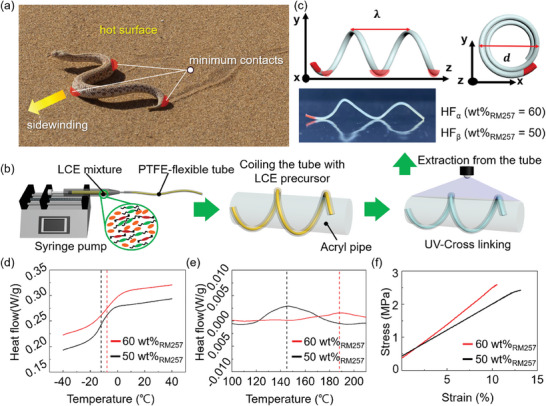
a) Image of a sidewinder snake with minimum contact on hot sands during lateral moving (a permanent credit of the image was purchased from 2023 iStockphoto LP). b) Illustration of the fabrication process to produce a LCE HF via templating and coiling of PTFE tube, followed by UV‐crosslinking and extraction of the HF. c) Illustration of the LCE HF with a definition of pitch (λ), a diameter (*d*), and a representative image of HF_α_ (*λ* = 3 cm and *d* = 7 mm). The red area represents the contact point of LCE HF with the underlying hot surface. DSC curves of LCEs with different RM257 weight percentages (60 and 50 wt.%), showing d) *T*_g_ and e) *T*_NI_. f) Stress–strain curves of LCEs with RM257 of 60 and 50 wt.%.

Soft robotics has emerged as a rapidly evolving field, offering environmental adaptation and autonomy for navigating challenging terrains and environments.^[^ ^1^, ^2^, ^3^, ^4^ ^]^ Compared with the conventional robots using rigid components, soft robots that utilize soft actuators are highly environmentally adaptive and deformable into different shapes for various environments and can be effectively miniaturized to the centimeter scale or smaller.^[^ ^5^, ^6^, ^7^ ^]^ In particular, they can find uses in complex, enclosed, or narrow spaces, such as gaps and vessels, and can navigate in maze‐like spaces.^[^ ^8^, ^9^, ^10^ ^]^ Among the diverse soft actuators, filament‐based ones have shown immense freedom moving in three dimensions, thus enabling complex locomotion that is not possible by film‐based actuators.^[^ ^11^, ^12^, ^13^, ^14^ ^]^ For example, helical structures have shown great promise as grippers,^[^ ^12^, ^15^, ^16^ ^]^ artificial muscles,^[^ ^17^, ^18^, ^19^, ^20^ ^]^ human‐robot interfaces,^[^ ^21^ ^]^ micro‐swimmers,^[^ ^22^, ^23^, ^24^ ^]^ and rolling robots.^[^ ^9^, ^25^, ^26^ ^]^ Among various stimuli‐responsive soft materials, liquid crystal elastomers (LCEs), with their remarkable anisotropy, have become highly sought‐after materials for soft actuators capable of fast, forceful, and reversible responsiveness.^[^ ^20^, ^27^, ^28^, ^29^, ^30^, ^31^, ^32^ ^]^ By controlling the orientation of liquid crystal (LC) mesogens and adopting diverse geometries, researchers have harnessed the programmability of LCEs to achieve efficient local and global motion and actuation.^[^ ^33^, ^34^, ^35^, ^36^ ^]^ The dynamic shape‐morphing of LCE actuators in response to external stimuli engenders sustainable movements, showcasing considerable potential in the design of self‐regulated soft robots that operate autonomously without the need for tethered or on‐board power and control systems.^[^ ^31^, ^37^, ^38^, ^39^ ^]^ Since the helical structures can reduce contact points with a substrate but a significant increase in weight‐to‐diameter ratio, LCE helices have shown a directional and self‐regulated rolling movement when exposed to light^[^ ^40^ ^]^ or heat.^[^ ^9^, ^41^, ^42^ ^]^ However, to date, helical or twisted LCE actuators only roll in a straight line that is usually normal to the long axis of filaments, similar to that of a simple LCE cylinder. This significantly limits the use of LCE helical structures for terrain navigation tasks where control of directional movement in 3D space is necessary. Unlike simple cylinders, helical or twisted LCE actuators can reversibly wind and unwind their structures.^[^ ^43^ ^]^ If we can take advantage of this reversible winding‐unwinding transformation as a controllable element of their motion, we can create rolling movements beyond the simple linear ones and traverse various paths. A recent study reported successful control over the rolling path of photo‐responsive LCE spiral ribbons through the spatial gradient of the optical stimulus.^[^ ^43^ ^]^ Nevertheless, very few literatures have explored the motion trajectories of LCE actuators for navigation of intricate terrains, and often it requires an externally controlled stimulus (e.g., light) to tune their trajectories step by step. Recently, Y. Zhao et al. reported a twisted LCE beam capable of maze escaping by rolling motion.^[^ ^44^ ^]^ The LCE beam is formed by twisting a rectangular beam, and the rolling mechanism is akin to the motion of a simple LCE cylinder. With the center of mass close to the ground, the movement is limited in the 3D space. Thus, the demonstrations of locomotion of LCE beam mainly focus on various configurations of 2D maze‐escaping. To enhance the versatility of self‐regulated soft robotics, comprehensive spatial explorations in 3D are desired. Sidewinder snakes, native to arid regions, employ a distinctive sidewinding motion with minimum contact points in the hot desert (**Figure** **1**a).^[^ ^45^ ^]^ Although the sidewinder snake does not actually roll in its movement, inspired by the sidewinder's form for survival, we develop a self‐adjusting, lateral‐rolling LCE helical filaments (HFs) soft robot that has minimal contacts on a given hot surface. By manipulating parameters such as the diameter and pitch of the HF, the modulus of LCE, and the temperature of the substrate, we direct the movement paths of the LCE HFs. Then, we demonstrate that LCE HFs are capable of autonomously exploring complex spaces with obstacles or finding a target in a large space over 600 cm^2^ by dynamically adjusting their rolling motions depending on the temperature of the underlying surface. In turn, the LCE HFs show to efficiently and autonomously navigate and overcome obstacles in maze‐like environments and staircases.

## 2 Results and Discussion

The LCE HF was fabricated using a flexible polytetrafluoroethylene (PTFE) tube (diameter, 900 µm) as the template (see the fabrication process in Figure 1b) with tunable pitch (*λ*, cm) and diameter (*d*, mm) (Figure 1c). The mesogenic mixture for LCE was prepared by mixing 4‐cyano‐4‐pentylbiphenyl (5CB) with reactive mesogens consisting of thiol‐terminated LC oligomers (LCOs) and reactive mesogenic monomers,1,4‐bis‐[4‐(6‐acryloyloxypropyloxy)benzoyloxy]−2‐methylbenzene (RM257). The weight percentages of RM257 with respect to the total mixture are 60 and 50 wt.%, respectively, leading to samples of different rigidity. The chemical structures of the mesogenic mixture can be found in Figure S1 (Supporting Information). The LCO was synthesized from 1,4‐bis‐[4‐(6‐acryl‐oyloxy‐hexyloxy)benzoyloxy]−2‐methylbenzene (RM82) and 1,3‐propanedithiol (1,3PDT), referred to as RM82‐1,3PDT via oxygen‐mediated step‐growth polymerization^[^ ^46^ ^]^ and the degree of polymerization (*n*) of LCO was ≈1 according to the quadrupole time‐of‐flight mass spectrometry(Q‐TOF‐MS/MS) spectrum (Figure S2, Supporting Information). Here, 5CB did not participate in polymerization but (i) lowered the viscosity of the precursor to facilitate infiltration of the precursors inside the tube and (ii) increased surface anchoring strength to assist the alignment of the precursor along the tube. To create the filament, we first injected the mesogenic mixture into the PTFE tube using a syringe pump at room temperature (25  C) where the mixture exhibited a nematic phase. The shear stress applied to the mixture induced the alignment of mesogens in the longitudinal direction of the tube (Figure S3, Supporting Information). Furthermore, to find the appropriate range of injection rate, we conducted a qualitative investigation of the alignment condition by varying the injection rate from 1 to 8 µL min^−1^ (Figure S4, Supporting Information). Based on the polarizing optical microscopy (POM) images, in our experiment, it was found that 3 µL min^−1^ represented the minimum injection rate for achieving uniaxial alignment along the long axis of the fiber. We investigated that the PTFE tube filled with the mesogen mixture was coiled around a transparent acryl pipe by 4π to have 2 helical pitches (*λ*) and the diameter (*d*) of HF was determined by the diameter of the acryl pipe. Due to the high flexural modulus of the PTFE template (≈1 GPa),^[^ ^47^ ^]^ the physically achievable minimum size of *λ* and *d* of HF via templating of the PTFE tube should be greater than ≈2 cm and ≈5 mm, respectively. On the other hand, when the values of *λ* and *d* of HF increase, there is an increase in rolling resistance due to a higher mass per unit contact point, which could interfere with the rolling motion. In fact, LCE HFs with *λ* > 3.5 cm and *d* > 12 mm did not roll well. Thus, in this study, we focused on the rolling motion of LCE HFs with *λ* in the range of 2.0–3.5 cm and *d* in the range of 5–12 mm. Then, the precursor of LCE HFs underwent photopolymerization under UV irradiation at 10 mW cm^−2^ for 30 min,^[^ ^31^, ^46^ ^]^ followed by removal of the PTFE tube. The resulting LCE HFs exhibited a helical shape preserved from the coiled PTFE tube (see inset photograph of HF with λ = 3 cm and d = 7 mm in Figure 1c). The helical shape remained after the removal of 5CB although the diameter of the HF slightly shrunk from 900 to 850 µm (Figure S5, Supporting Information). Although there might be voids after the removal of 5CB, we did not observe them in the scanning electron microscopy (SEM) image (Figure S6, Supporting Information), which was consistent with our prior work.^[^ ^48^, ^49^ ^]^ This could be attributed to the soft nature of LCE, causing the pores to collapse after drying due to large surface tension. In addition, to confirm the details of mesogenic orientation, we conducted a small and wide‐angle X‐ray scattering (SWAXS) using a straight filament of the LCE prepared from the precursor of HF_α_ with the same procedure used to produce HFs. SWAXS results confirmed that mesogens were well‐aligned in parallel along the long axis of the filament (Figure S7a, Supporting Information). The intermolecular spacing was ≈4.58 Å, consistent with the typical π−π stacking distance between mesogens.^[^ ^31^ ^]^ Furthermore, the degree of orientation was quantitatively analyzed using the Hermans orientation parameter,^[^ ^50^ ^]^ S, calculated from the azimuthal intensity profile at *q* = 1.39 Å^−1^ (Figure S7b, Supporting Information). The calculated value, S ≈ 0.36, indicates that the mesogens of LCE were fairly well‐oriented along the longitudinal direction of the LCE filament. Both LCEs with different RM257 mass concentrations, 60 and 50 wt.%, had glass transition temperatures (*T*_g_) of − 8.4 and − 11.8  C, respectively (Figure 1d), and nematic to isotropic transition temperatures (T_NI_), ≈188.3 and ≈143.1  C, respectively, as measured by differential scanning calorimetry (DSC) (Figure 1e). Thus, LCE HFs were in a rubbery state at room temperature. The Young's moduli of the LCEs with 60 and 50 wt.% of RM257 were determined at 10% strain (in the elastic regime) as 31.25 and 24.93 MPa, respectively, from the stress–strain curves in the straightened forms of HFs under a uniaxial strain at room temperature (Figure 1f). To avoid errors in property measurements due to the unraveling of the helical structure during the strain, we conducted experiments using straight filaments of the LCEs prepared from the precursor of HF_α_ and HF_β_. In addition, the straight filaments exhibited maximum uniaxial contractions of 30% and 40%, respectively (Figure S8, Supporting Information).

**Figure 2:**
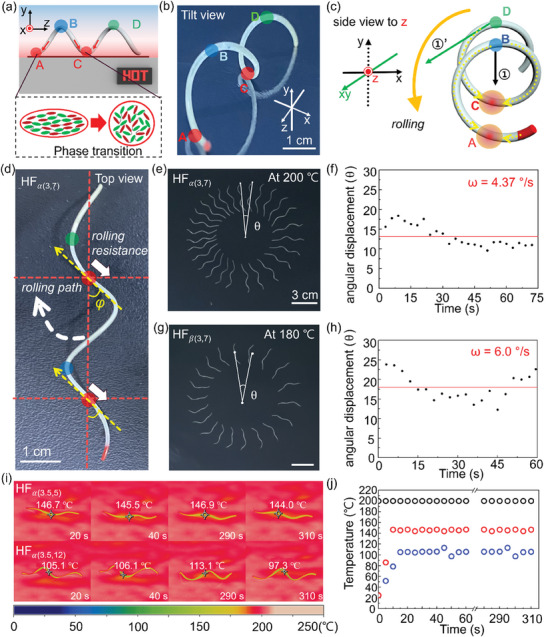
a) Schematic illustration of phase transition of a LCE HF from nematic to isotropic state at the contacts, A and B on a hot surface. b) The optical image of HF_α(3,7)_ in a tilt view. c) Schematic illustration of the rolling mechanism of HF via thermal contraction at the contact points. d) Top view of HF_α(3,7)_, representing the direction of rolling (white dashed arrow) and rolling resistance (white solid thick arrows), where φ defined as helix angle. e,g) Overlaid images of the HF_α(3,7)_’s and HF_β(3,7)_’s rolling at 200 and 180  C, respectively showing circular arc trajectories. Scale bars: 3 cm. f,h) the corresponding angular displacements of HF_α(3,7)_ and HF_β(3,7)_ as a function of time. i) IR camera images of HF_α(3.5,5)_ and HF_α(3.5,12)_ on the hotplate (200  C) during their rolling. j) The corresponding temperatures of HFs as a function of time (black, red, and blue circles indicate hotplate, HF_α(3.5,5)_ and HF_α(3.5,12)_, respectively).

From now on, the HF produced from each LCE precursor is referred to as HF_α_ (60 wt.% of RM257) and HF_β_ (50 wt.% of RM257), respectively (Figure 1c). For HF_α_ or HF_β_ with different λ (cm) and d (mm), we will refer to them as HF_α(λ, d)_ or HF_β(λ, d)_. When LCE HF_α(3,7)_ or HF_β(3,7)_ was placed on a hotplate above the respective T_NI_ (≈200 or 180  C, respectively), they rolled autonomously and continuously driven as the HFs made the dynamically changing thermal contacts with the hotplate surface (Movie S1, Supporting Information). To understand the rolling mechanism of the LCE HFs, we analyzed the shape‐changing process using the HF_α(3,7)_ upon hot surface contact. When HF_α(3,7)_ was placed on the hotplate above *T*_NI_ = 200  C, it made contacts onto the hot surface, denoted as points “A” and “C”, respectively, (**Figure** **2**a). Figure 2b shows the corresponding photograph of the HF_α(3,7)_ placed on the hotplate in a tilted view. At ≈200  C, HF_α(3,7)_ contracted around A and C in the direction of the HF_α(3,7)_’s rotating axis as denoted with yellow dashed lines in Figure 2c. Here, B and D represent the highest points of the HF_α(3,7)_, located at half of a circle away from A and C, respectively. Figure 2c schematically illustrates the mechanism by which the HF_α(3,7)_ gains momentum as it starts rolling. Due to contraction at points A and C, point B experienced a pulling force in the opposite downward spiral direction, as indicated by the black solid arrow with “①”. Consequently, point B moved vertically toward the ground (as shown by the black arrow labeled with ① in Figure 2c). Meanwhile, point D experienced a force pulling it toward contact point C. As a result, the posterior part of the HF, including point D, tilted to the left in the yz plane (with reference to Figure 2c) as indicated by the green arrow label with ①'. This movement of point D caused the center of mass of the HF to shift toward the yz plane as denoted by the yellow arrow in Figure 2c, resulting in the rolling momentum being generated in the same direction as D for the portion, including point B. Similar to cylinders or helical actuators previously reported in literatures that responded to light or thermal stimuli and exhibited self‐regulating rolling movements,^[^ ^40^, ^41^ ^]^ our HF_α(3,7)_ demonstrated autonomous motion based on continuous thermal self‐regulation, wherein the heat exchange occurred as the HF_α(3,7)_ underwent dynamical thermal contacts during rolling. However, an intriguing difference between our study and from literatures^[^ ^9^, ^41^ ^]^ was evident when overlaying the HF rolling images taken at 3 s intervals (Figure 2e,g). Both HF_α(3,7)_ and HF_β(3,7)_ did not roll in a straight path but followed a curved trajectory. That is, our HFs rolled in the direction driven by the inertial motion of the rolling with the reversible thermal strain imposed along the helical rotation in contact with the substrate. The inertial motion generates a curved path in the shape of an arc tangent to the helical rotation, depending on the helix angle *φ* denoted in Figure 2d, indicating the inclination between the helix's axis and its helical rotation. The motion is due to the resistance to rolling formed in the opposite direction of the helix's rotational direction.^[^ ^40^, ^51^ ^]^ As suggested,^[^ ^40^, ^43^ ^]^ when the helical angle is close to ≈45°, the synergistic effect of rolling resistance and helical geometry produces a circular arc trajectory. In order to show the congruence between our HFs and the general principle that underlies helix's rolling, we performed experiments utilizing a toy model analogous to our HFs. As demonstrated in Movie S2 and Figure S9a (Supporting Information), when the helical toy model with *φ* = 45° rolled down a 10° incline, it displayed a curved rolling path in the direction tangent to the surface opposite to the rolling resistance. The moving path was highly dependent on *φ*. As *φ* decreased, the roll path became more linear as shown in the latter part of Movie S2 and Figure S9b (Supporting Information), with *φ* = 18°. Here, the motion of the object aligned more closely with the direction of inertial motion. In the next section, we will discuss in more detail of the path changes as a function of *φ*. The measured average angular rates (ω) for 25 steps in a single round of rolling ω = θ(°)/time(s) were 4.37 and 6.0 ° s^−1^ for HF_α(3,7)_ and HF_β(3,7)_, respectively (Figure 2f,h). *θ* represents the angular displacement at 3s intervals according to Figure 2e,g. Furthermore, we examined the rolling behavior of HF_α(3,7)_ with different chirality, one coiled clockwise and the other counterclockwise (Figure S10, Supporting Information). Following the rolling principle described in Figure 2c,d, depending on the helical rotation direction in contact with the substrate, two HFs of opposite chirality exhibited opposite directions of rolling paths. For the clockwise‐coiled HF_α(3,7)_ (Figure S10a,b, Supporting Information), the helical rotation in contact with the substrate is ≈45° away from the helical axis, as denoted by red arrows, exhibiting rolling in a clockwise direction with a curved path. In contrast, for the counterclockwise‐coiled HF_α(3,7)_ (Figure S10c,d, Supporting Information), the helical rotation was ≈135° away from the helical axis, as denoted by red arrows, exhibiting rolling in a counterclockwise direction with a curved path.

HF_β(3,7)_ appeared to roll slightly faster than HF_α_, which could be attributed to the slightly softer elasticity of HF_β_, allowing for a wider range of contraction‐relaxation than HF_α_. Indeed, as observed in Figure 2e,g, at the same 3s intervals, the angular displacement (radian) of HF_β_ was wider than that of HF_α_. The rolling along the curved path was highly autonomous and sustainable due to the self‐regulation of the heat flux, with the top and bottom of HF being cooled and heated, respectively. As compared with helical, twisted or torsional LCE structures reported in literature,^[^ ^9^, ^41^ ^]^ our HFs were spring‐like helices with large air volume fraction within the helix (e.g., 98.6% when *d* = 10 mm and λ = 3 cm), which could act as reservoir for maintaining the continuity of the self‐regulated rolling during the heating and cooling cycles. To confirm its role, we used an infrared (IR) camera to monitor the temperature changes of the two HFs, HF*_α_*_(3.5,5)_ and HF*_α_*_(3.5,12)_, on the same hotplate at 200  C during rolling. As seen from Figure 2i,j and Movie S3 (Supporting Information), the temperatures of both HFs were lower than that of the surface of the hot plate (Figure S11, Supporting Information); they remained almost constant at ≈145 and 107  C, respectively, throughout the duration of the rolling. As expected, HF*_α_*_(3.5,12)_ with a larger diameter had a lower body temperature due to a longer distance away from the hot surface and more air stored in its body. Remarkably, the HF*_α_*_(3.5,12)_ could continuously roll for more than an hour on the hotplate at 200  C in ambient air (≈25 °C) (see Movie S4, Supporting Information).

**Figure 3:**
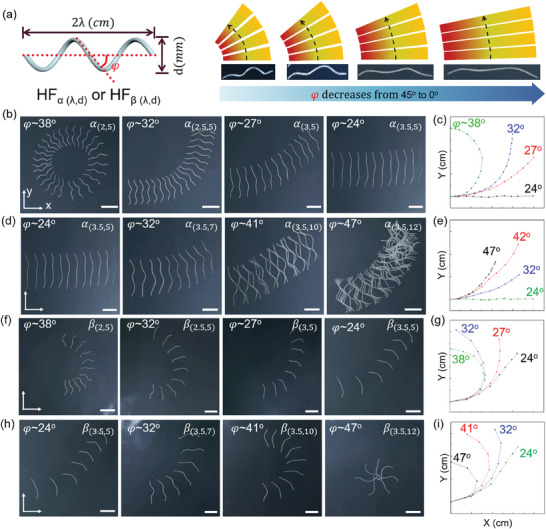
a) Description of the helical angle, φ(^o^), defined in LCE HF and Illustration of HF's rolling motion change with respect to φ. The inset images are HF_α(2,5)_, HF_α(2.5,5)_, HF_α(3,5)_ and HF_α(3.5,5)_, respectively. Overlaid images of (b,d) HF_α_ and (f,h) HF_β_ with a variation of φ, showing the path variation during the self‐rolling at 200 and 180  C, respectively. The corresponding trajectories of (c,e) HF_α_’s and (g,i) HF_β_’s rolling in the xy plane. All scale bars: 3 cm.

In general, the rolling path of a helix is the result of the interplay between the helix angle *φ* and rolling resistance. *φ* governs the efficiency of rolling.^[^ ^40^ ^]^ Since *φ* can be determined by *λ* and *d* of LCE HFs, HF*_α_* or HF_𝛽_ with different *φ* will be expressed as HF_α(λ,d)_ or HF_β(λ,d)_ (**Figure** **3**a). As shown in Movie S2 (Supporting Information), when *φ* was close to 0°, the helix's axis was nearly parallel to the rolling direction. Thus, rolling resistance was significantly subdued, resulting in a nearly linear rolling path. As *φ* increased to be close to 45°, the helical structure imparted a component of motion that was directed to the helix's axis, in proportion to the increase in φ. As *φ* continued to increase, this directional motion became more and more dominant, causing HFs to veer away from a linear path. Now we examined the movement paths of HFs as a function of *φ* at a constant hotplate temperature, where *φ* is a function of *λ* and *d* of HF (see Figure 3a; Figure S12, Supporting Information) as

(1) $$tan^{-1}\left(\frac{\pi d}{\lambda }\right)=\varphi$$

Here, *λ* ranged from 2.0 to 3.5 cm and *d* ranged from 5 to 12 mm. Accordingly, φ ranged from ≈47° to 24°. Figure 3b,d,f,h shows the overlaid images captured at 3s intervals of the rolling motion of HF*_α_* and HF*_β_*, respectively, with varying *λ* and *d*. Figure 3c,e,g,i provides corresponding traces of these paths in the xy‐plane for HF*_α_* and HF*_β_*, respectively. As φ decreased, the trajectory shifted from curvilinear to linear rolling (see Figure 3b–i). Overall, HF*_β_* exhibited more pronounced irregular paths such as sharp turns. This is possibly because of its softer body and larger thermal contraction compared with HF*_α_*, leading to higher energy loss and increased rolling resistance.

**Figure 4:**
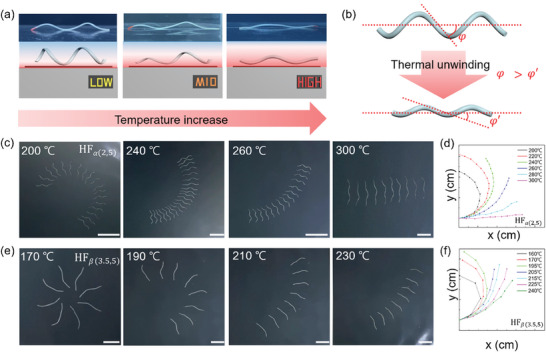
a) Schematic illustration of the LCE HFs on a hotplate at different temperatures and the corresponding side view images of HF_α(2,5)_ at 200, 260, and 300  C during rolling. b) Illustration of helical angle change of the HF through thermal unwinding as temperature increases. c,e) The overlaid images of HF_α(2,5)_ and HF_β(3.5,5)_ during rolling at different temperatures. d,f) The corresponding path variation of the HF_α(2,5)_ and HF_β(3.5,5)_ in the xy‐plane. All scale bars: 3 cm.

Next, we investigated the rolling paths as a function of the substrate temperature. As seen in **Figure** **4**a, when the axial contraction in the direction of helical rotation surpassed its relaxation capacity, the helix gradually unwound during rolling, resulting in a reduction of *φ* due to axial contraction (Figure 4b). For the generality of our system, we used different sizes of HFs in this part, HF*_α_*_(2,5)_ and HF_𝛽(3.5,5),_ from previous HFs in Figure 3. Figure 4c,e presents the overlaid images, at 3s intervals, of HF*_α_*_(2,5)_ and HF_𝛽(3.5,5)_ at temperatures above each T_NI_, ranging from 200 to 300  C and 170 to 230  C, respectively. Figure 4d,f presents the trajectories of HF*_α_*_(2,5)_ and HF_𝛽(3.5,5)_, respectively, at each temperature in the xy‐plane. It was clear that as the temperature increased, the movement trajectory became closer to a straight line due to the growing helical unwinding. Notably, these results highlight the potential of a single HF system to dynamically alter its paths depending on the variations of the thermal stimulation. When the temperature was significantly higher than T_NI_, the axial contraction of LCE HF became overwhelmingly dominant over its relaxation, leading to a sharp decline in the reversible axial contraction‐relaxation efficiency. Consequently, the lifetime of HF rolling decayed, and the propulsive force for rolling was lost. Therefore, further development of LCEs with a lower operating temperature and a broader range of thermal responsiveness will be desired.

**Figure 5:**
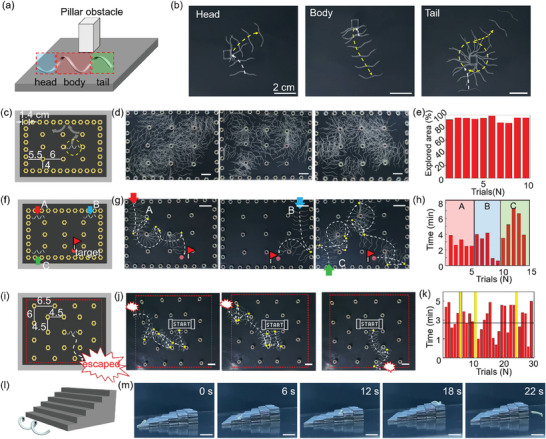
a) Sketch of LCE HF presenting three different parts, head, body, and tail, and a pillar obstacle. b) The overlaid images of HF*_α_*_(3,7)_, showing path variation before and after collision with different parts of the obstacle at 190  C. White and yellow arrows indicate the locomotion pathway before and after collision with the pillar, respectively. Scale bars: 2 cm. c) Sketch of the confined terrain with nut‐like obstacles. d) The overlaid images of the self‐exploration of HF*_α_*_(3,7)_’s rolling in the corresponding terrain at 190  C for 20 min. All Scale bars: 3 cm. e) The self‐explored area by HF*_α_*_(3,7)_ for 10 trials. f) Schematic illustration of the terrain for a target finding. g) The overlaid images of target finding of HF*_α_*_(3,7)_’s rolling from three different starting points, A, B, and C, respectively, at 190  C. Scale bars: 3 cm. h) The target‐finding time as a function of trials from different starting points. i) Schematic illustration of the maze escaping of LCE HF*_α_*_(3,7)_. j) The overlaid images of the maze escaping of HF*_α_*_(3,7)_’s rolling in the corresponding maze at 190  C. The scale bars: 3 cm. k) Maze escaping time as a function of trials. l) Illustration of overcoming a staircase‐like obstacle of HF’ rolling. m) The images show overcoming the staircase‐like obstacle of HF*_α_*_(3,7)_ at 210  C.

Due to their deformability, lightweight, and flexibility, soft robots outperform rigid robots when navigating diverse environments, in particular those with challenging settings.^[^ ^52^ ^]^ In an intricate space with obstacles, straight paths may lead to a collision and are often repetitive around the obstacles. In contrast, a curved trajectory of the soft robot excels in avoiding obstacles, enhancing safety and efficiency. Therefore, we evaluated our LCE HF system for sustained and nonlinear movement using HF*_α_*_(3,7)_ on hot surfaces in constrained spaces with various obstacles. First, we observed a rolling motion of a single HF*_α_*_(3,7)_ around a glass pillar on the hot plate ≈190  C (**Figure** **5**a,b; and Movie S5 and Figure S13, Supporting Information). HF*_α_*_(3,7)_ changed its path after collision with the pillar, denoted as white dashed arrows for the pre‐path change and yellow dashed arrows for the post‐path change based on its head (inner side of the curved trajectory), body (middle part), and tail (outer side of the curved trajectory) of HF*_α_*_(3,7)_. When the head or tail encountered obstacles, it often showed behaviors such as lingering and circling around the obstacle, followed by snapping and changing its path in a different direction and continuing to roll. When part of the body of HF*_α_*_(3,7)_ collided with the obstacles, it briefly circled around without rotation, then transitioned its path in the opposite direction of the previous trajectory and rolled. These results were in good agreement with the snapping behaviors observed from helicoidal LCE ribbons.^[^ ^9^ ^]^ When HF*_α_*_(3,7)_ encountered the obstacle, the rolling was hindered, and more elastic strain energy was stored in the HF due to the thermal contraction‐induced unwinding of HF, which was momentarily released upon snapping.

Next, we evaluated the terrain exploration ability, target finding, and maze escape by HF*_α_*_(3,7),_ and all experiments were conducted on the hot plate ≈190  C. Since HF_β_ exhibited softer properties, resulting in more irregular patterns in thermal motion compared to HF_α_, we primarily used HF_α_ for the experiment on terrain explorations. As shown in Figure 5c and Movie S6 (Supporting Information), HF*_α_*_(3,7)_ was put in a space containing nut‐like hexagonal columns spaced a few centimeters apart (with a total surface area of ≈600 cm^2^), to determine how quickly and how much the area it could explore. Figure 5d depicts overlaid snapshots of HF*_α_*_(3,7)_’s rolling motion captured at 3s intervals for the 1st, 5th, and 10th trials. In all 10 trials, the HF*_α_*_(3,7)_ explored over 90% of the surface area within 20 min (Figure 5e). We then conducted 15 trials of target finding task, where the HF*_α_*_(3,7)_’s rolling motion was initiated at different starting points (labeled as A, B, and C) within the same space (Figure 5f; Movie S7, Supporting Information). The goal was to determine how quickly HF could reach a specific target in the confined space, where the target was the nut marked by red color labeled with a red flag. Although minor variations were observed depending on the starting positions, the average time taken to find the target was ≈4 min (Figure 5h). Figure 5g provides an overlaid image of snapshots at 3‐second intervals, showing the paths taken by the HF*_α_*_(3,7)_ from points A, B, or C to the target. It was clear that our HFs could locate a target when exploring a specific space. Encouraged by the results, we assessed how quickly HF could exit a maze‐like space containing the arranged nuts, as depicted in Figure 5i. The rolling motion of HF*_α_*_(3,7)_ was initiated from the central area (indicated by the “start” label). As illustrated in Figure 5k, following 30 trials, HF_α(3,7)_ managed to escape in under 3 min. on average. This was notably faster than the 20 min. reported in the literature for escaping a maze with a single obstacle,^[^ ^9^ ^]^ despite the increased complexity of the maze in this experiment, which included 21 obstacles. In comparison, our LCE HF can escape a maze with more than 15 times higher area complexity (*C* = 150 N m^−2^), defined as the number of obstacles (*N*) per overall area (m^2^), compared to the literature value (*C*_area_ = 10 N m^−2^).^[^ ^9^ ^]^ Figure 5j presents the overlaid images of HF_α(3,7)_’s escaping the maze at 3s intervals for the 8th, 16th, and 29th trials from the 30 trials. In this experiment, we observed that HF_α(3,7)_ selected different random paths in each attempt, but interestingly, they all had more or less similar time to escape (all within 10 min), implying consistency of our system terrain navigation. Furthermore, the curve‐path navigation of HF_α(3,7)_ allowed it to successfully perform maze escape in other complex spaces such as zigzags, vortexes, and intersecting U‐shaped terrains (Figure S14 and Movie S8, Supporting Information).

Lastly, we explored HF's capability to ascend 3D staircase‐like obstacles using HF*_α_*_(3,7)_ (Figure 5l,m; Figure S15 and Movie S9, Supporting Information). As seen in Figure S15 (Supporting Information), HF*_α_*_(3,7)_ could surmount obstacles with heights up to 60% of its diameter (≈7 mm) through a rolling motion on a six‐step staircase at 210  C (Figure 5m). Each step had a height of ≈2 mm, achieved within the operation temperature ranging from 170 to 250  C. We note the T_NI_ is ≈188.3  C (Figure S15c, Supporting Information). Below ≈170  C or above 250  C, HF*_α_*_(3,7)_ was not able to overcome the stairs, implying that a highly flexible twisting and untwisting of HF*_α_*_(3,7)_ well above T_NI_ to store and release the elastic energy within HF*_α_*_(3,7)_ is the key to enable successful traversal of the stairs.^[^ ^51^ ^]^ Below 170  C, HF*_α_*_(3,7)_ did not roll because the temperature is below T_NI_, whereas above 250  C, HF*_α_*_(3,7)_ may have permeant damage due to the overwhelming inflow of heat into the HF.

## 3 Conclusion

we prepare a self‐adjusting, lateral‐rolling soft robot from LCE HFs with spring‐like helical structures inspired by the sidewinder snake's unique shape on a hot surface. By harnessing the reversible thermal contraction and expansion along the long axis of LCE filament whereas the air in‐between the spring serves as the reservoir for cooling, we have successfully demonstrated various curvy pathways of the rolling movements regulated by parameters including the HF's diameter, pitch, Young's modulus, and the hotplate temperature. In turn, we demonstrate LCE HFs as self‐rolling soft robots navigating challenging terrains using HF*_α_*_(3,7)_. In maze‐like spaces, HF*_α_*_(3,7)_ explores over 90% of a surface (≈600 cm^2^ surface area) with nut‐like obstacles in 20 min, finds a target in 4 min, and escapes the maze in 3 min. Moreover, it could climb staircase obstacles of height 60% of its helical diameter via rolling. These results clearly illustrate the potential of LCE HFs as autonomous soft robots performing tasks such as navigation in complex environments, obstacle avoidance, target detection, and rapid maze escape. We believe that the development of a self‐adjusting lateral‐rolling soft robot based on helical structures presents one of the innovative advancements in versatile and adaptable robotic locomotion strategy for the field of terrain exploration.

## 4 Experimental Section

### Materials

RM82, RM257 and 5CB were purchased from Henan Wentao Chemical Product Co. Ltd. 1,8‐diazabicyclo[5.4.0]undec‐7‐ene (DBU; 98%), 1,3PDT (>99%), 2,2‐dimethoxy‐2‐phenylacetophenone (DMPA; 99%) were purchased from Sigma–Aldrich. Dichloromethane (DCM), Ethanol, Sodium chloride (NaCl), and Hydrochloric acid (HCl; 36.5–38.0%) were purchased from Duksan Pure Chemical. Magnesium sulfate (MgSO_4_; anhydrous powder) was purchased from Samchun Pure Chemical. Butylhydroxytoluene (BHT; analytical standard) was purchased from Tokyo Chemical Industry Co. (TCI).

### Preparation of the LCE Precursors

RM82‐1,3PDT (LCO) was first synthesized according to the literature through step‐growth polymerization of RM82 and 1,3PDT.^[^ ^46^ ^]^ To prepare the LCE precursors, RM257 and LCO were mixed in a weight ratio of 1.5:1 and 1:1, referred to as the HF*_α_* and HF*_β_*, respectively, consisting of 60 and 50 wt.% of RM257, respectively, in the mixture. After that, 30 wt.% 5CB with respect to the mixture of LCO and RM257 was added, followed by the addition of 2 wt.% DMPA with respect to the total mixture of LCO, RM257, and 5CB was added as a photo‐initiator. The LCE precursor was obtained after magnetic stirring at 100  C for an hour to completely mix the materials and cool down to room temperature (25  C).

### Preparation of LCE HFs

The LCE HFs were prepared by injecting the LCE precursor into the PTFE flexible tubing (Masterflex Transfer tubing) using a syringe pump with thickness between inner and outer tube, ≈1.0 mm, (KDS Legato 100, single syringe infusion pump) in rate ranges of 1–8 µL min^−1^. The PTFE tubing was coiled onto a glass rod (outer diameter, 3 mm) or acryl pipes (out diameters, 5, 8, 10 mm) with variable pitches (2.0–3.5 cm), followed by photopolymerization under 365 nm UV light exposure (Ushio Shenzhen, Inc.) with 10 mW cm^−2^ for 30 min. The PTFE tubing was removed and the LCE filaments were immersed in ethanol for 24 h to remove non‐reacted materials, including 5CB and residual monomers and oligomers.

### Characterization of LCE HFs and the Locomotion of LCE HFs

The nematic to isotropic phase transition temperature (*T*_NI_) and the glass transition temperature (*T*_g_) of LCE HFs were measured by differential scanning calorimetry (DSC) (Discovery DSC 25, TA instrument). The mass of LCO was measured by quadrupole time‐of‐flight mass spectrometry (Q‐TOF‐MS/MS, maXis‐HD, Bruker) to determine the degree of polymerization (*n*), where the mass spectrum was collected using atmospheric pressure Chemical Ionization (APCI). Samples were placed on aluminum hermetic crucibles and scanned at two cooling and heating cycles of from −50 to 220  C with a rate of 10  C min^−1^ under nitrogen gas (N_2_) conditions. The data from the second cycle were reported here. Alignment of LC mesogens to the longitudinal direction within the PTFE tubing was characterized under optical microscopy (LV100POL, Nikon) with crossed polarizers using NIS elements software. All digital images and videos were taken by iPhone 11Pro. The thermal actuation of LCE HFs was observed onto the hotplate (HPR‐4030, AS ONE) that was covered with a commercially available high‐temperature‐resistant PTFE‐coated glass‐fiber sheet (Roband Ltd.). The covered PTFE film shows a considerably flat roughness as seen in the SEM image (Figure S16, Supporting Information). The mechanical properties of LCE HFs were measured using the universal testing machine (H1KT machine, Tinius Olsen Ltd.). IR images of LCE HFs were taken by spot finder IR camera (Xi 400, Optris). X‐ray scattering experiments were performed on the Dual‐source and Environmental X‐ray Scattering (DEXS). A Xeuss 2.0 with a GeniX3D S4 source (Cu Kα, *λ* = 1.54 Å) and a PILATUS3 1 m detector (981 × 1043 pixels, pixel dimension 172 µm) was used with the sample‐to‐detector distance of 16 cm. The LCE filament is placed in the perpendicular direction relative to the beam direction in a transmission configuration.

## Conflict of Interest

The authors declare no conflict of interest.

## Author Contributions

D.S.K. and S.Y. conceived the ideas. D.S.K. and Y.B.K. fabricated samples and collected data. D.S.K., Y.B.K., and S.Y. analyzed the results. D.S.K., Y.B.K., and S.Y. wrote the manuscript. D.S.K. and S.Y. supervised the research. All authors discussed the results. S. Y. and D.S.K. are co‐corresponding authors for the research.

## Supporting information

Supporting Information

Supplemental Movie 1

Supplemental Movie 2

Supplemental Movie 3

Supplemental Movie 4

Supplemental Movie 5

Supplemental Movie 6

Supplemental Movie 7

Supplemental Movie 8

Supplemental Movie 9

## Data Availability

The data that support the findings of this study are available from the corresponding author upon reasonable request.
