# Supplementary material for: Sidewinder‐Inspired Self‐Adjusting, Lateral‐Rolling Soft Robots for Autonomous Terrain Exploration
Source: Adv Sci (Weinh). 2024 Jan 29;11(14):2308350. doi: 10.1002/advs.202308350 (PMC11005722; doi:10.1002/advs.202308350)
Supplement: Supplementary file 1 — Supporting Information [file ADVS-11-2308350-s007.pdf]

ADVANCED SCIENCE
Open Access

Supporting Information

for *Adv. Sci.*, DOI 10.1002/advs.202308350

Sidewinder-Inspired Self-Adjusting, Lateral-Rolling Soft Robots for Autonomous Terrain Exploration

Young Been Kim, Shu Yang and Dae Seok Kim**

Supporting Information

Sidewinder-inspired self-adjusting, lateral-rolling soft robots for autonomous terrain exploration

Young Been Kim, Shu Yang, Dae Seok Kim**

Y. B. Kim, D. S. Kim

Pukyong National University, Department of Polymer Engineering, 45 Yongso-ro, Nam-gu, Busan, 48513, S. Korea

E-mail: daeseok@pknu.ac.kr

S. Yang

Department of Materials Science and Engineering, University of Pennsylvania, 3231 Walnut Street, Philadelphia, PA 19104, USA

E-mail: shuyang@seas.upenn.edu

This Supporting Information includes:

SI Figures and captions (Figure S1-S16)

Captions for Supporting Movies (Movie S1-S9)

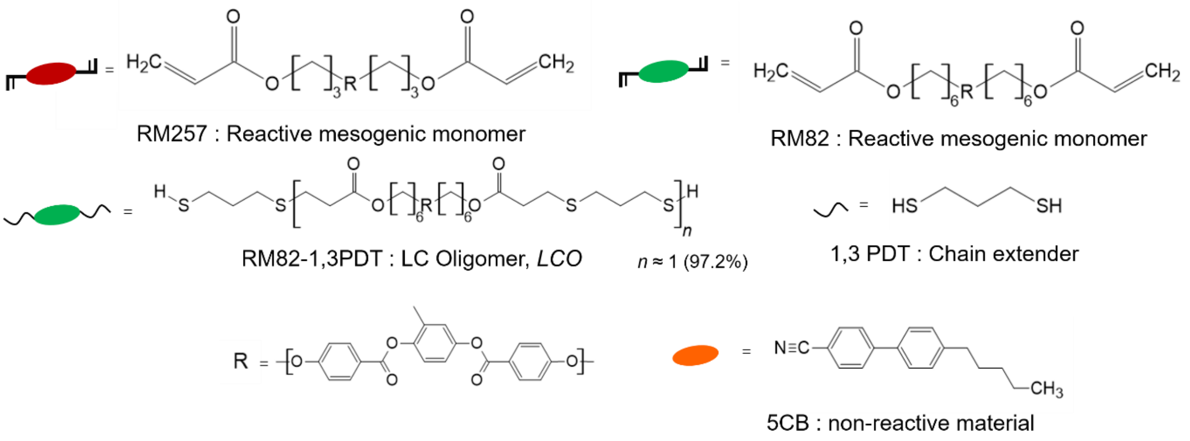

Figure S1. Molecular structures of the reactive mesogenic monomer (RM257, RM82), chain-extender (1,3PDT), non-reactive mesogenic molecule (5CB), and LC oligomer (RM82-1,3PDT). The degree of oligomerization is n≈1 (97.2%) measured by Q-TOF-MS/MS spectroscopy.

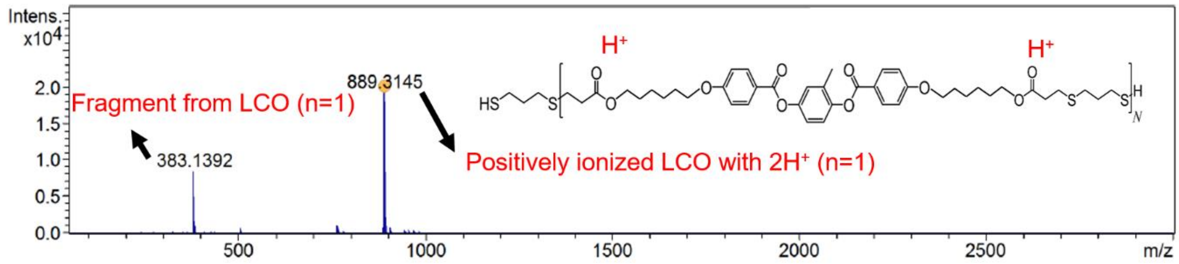

Meas. m/z	#	Ion Formula	m/z	err [ppm]	mSigma	# mSigma	Score	rdb	e⁻ Conf	N-Rule
889.3145	1	C45H61O10S4	889.3142	-0.3	3.2	1	100.00	15.5	even	ok

Figure S2. Q-TOF-MS/MS spectrum of the LCOs collected using atmospheric pressure chemical ionization (APCI).

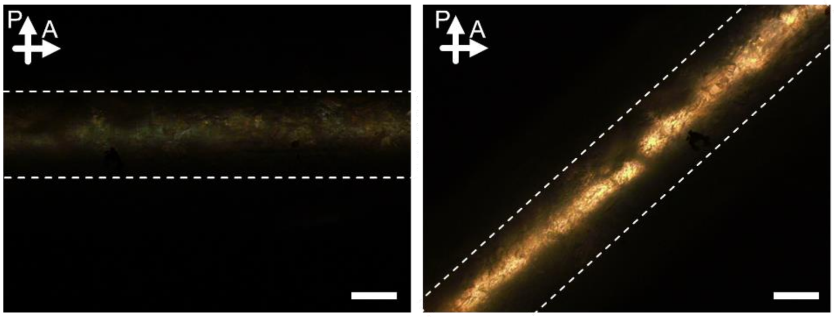

Figure S3. Polarized optical microscope (POM) images of the nematic precursor of LCE HF_{α} in the PTFE tube with and without a 45° rotation of the sample, showing alignment along the longitudinal direction of the tube (white dashed lines indicate borderlines of PTFE tubes). Scale bars: 500 μm.

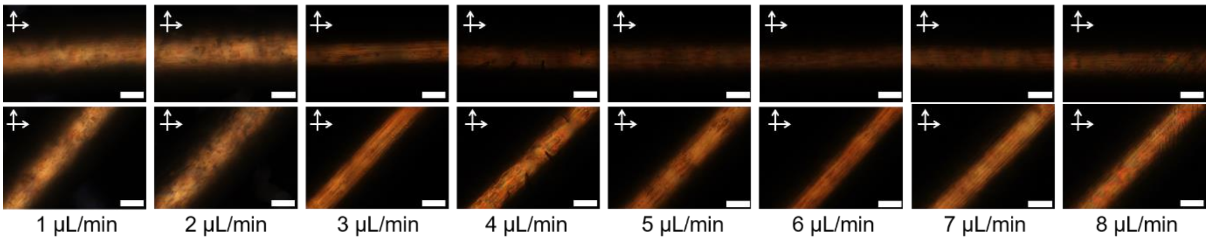

Figure S4. POM images of the LCE HF_{α} prepared with different injection rates of the LCO mixtures without (top panels), and with (bottom panels) a 45° rotation of the samples. Arrows indicate the crossed polarizers. Scale bars: 500 μm.

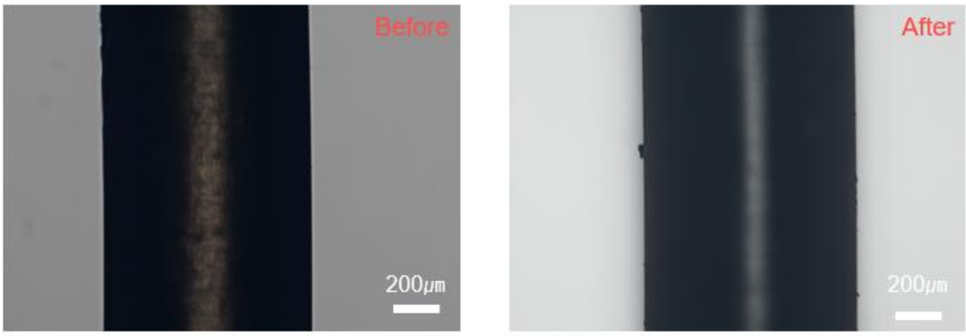

Figure S5. Optical microscopy images of the LCE HF_{α} before and after removal of 5CB and unreacted residuals.

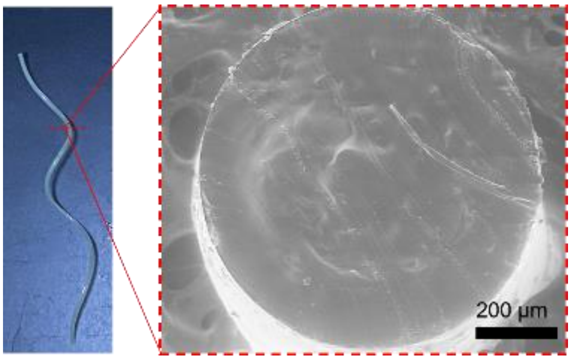

Figure S6. Cross-sectional SEM image of the LCE filament, $HF_{\alpha(3,7)}$.

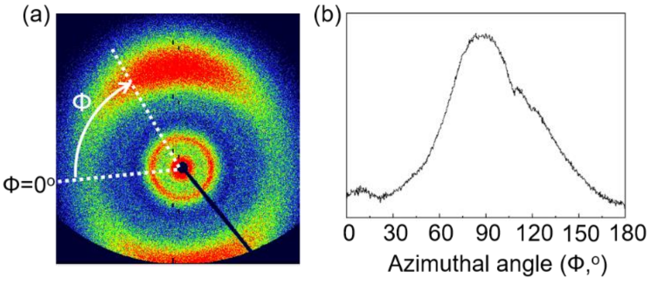

Figure S7. (a) Two-dimensional X-ray diffraction images of the straight LCE filament. (b) Azimuthal intensity profiles at q = 1.39 $Å^{-1}$ of the straight LCE filament.

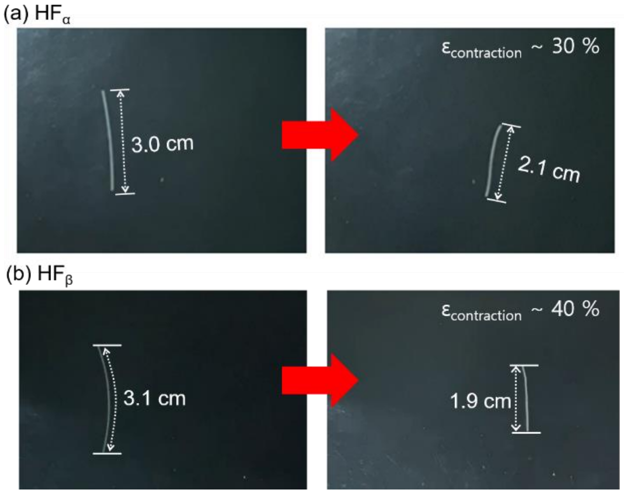

Figure S8. Photographs of the straight LCE filaments produced from LCE precursors of (a) HF_{α} and (b) HF_{β}, respectively, before and after the thermal contraction at 300 °C.

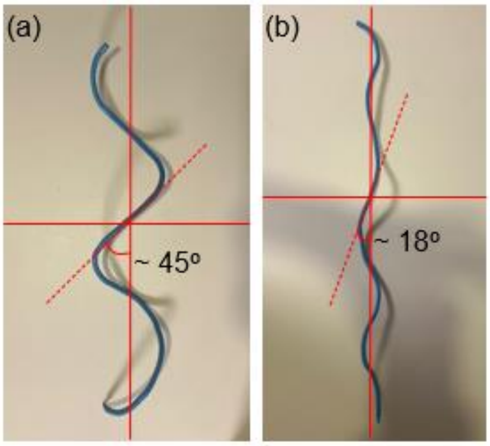

Figure S9. A helical toy model of different helical angles. The one with a helical angle of (a) ~45º shows a curved rolling path whereas that of (b) ~18º shows a straighter rolling path.

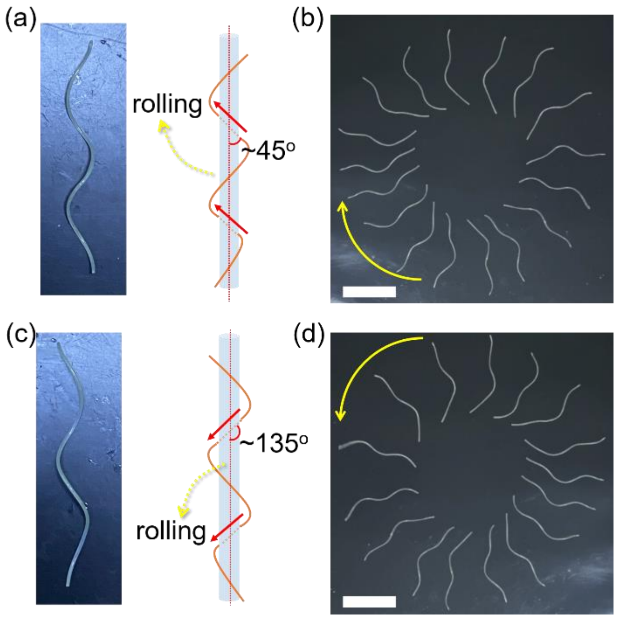

Figure S10. The rolling behaviors of $HF_{\alpha(3,7)}$ with opposite chirality. (a,c) The clockwise- and counterclockwise-coiled $HF_{\alpha(3,7)}$ and the corresponding schematic illustrations. (b,d) The overlaid images of the rolling of the $HF_{\alpha(3,7)}$ that are coiled in the clockwise and counterclockwise directions, respectively, upon heating at 200 ℃, exhibiting different circular arc trajectories. Scale bars: 3 cm.

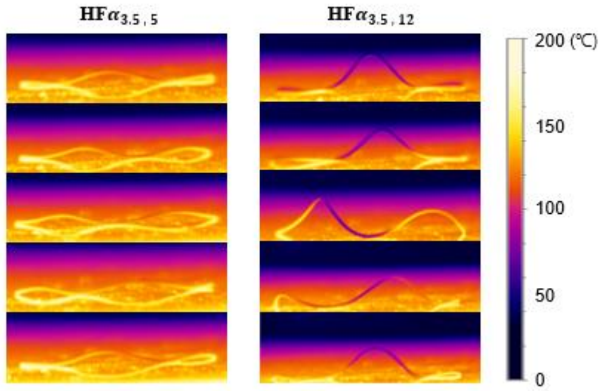

Figure S11. Side-view IR images showing the temperature gradient of HF_{α} of the same pitch (3.5 cm) but different diameters (5 mm and 12 mm) during rolling locomotion. The hotplate temperature is 200 °C.

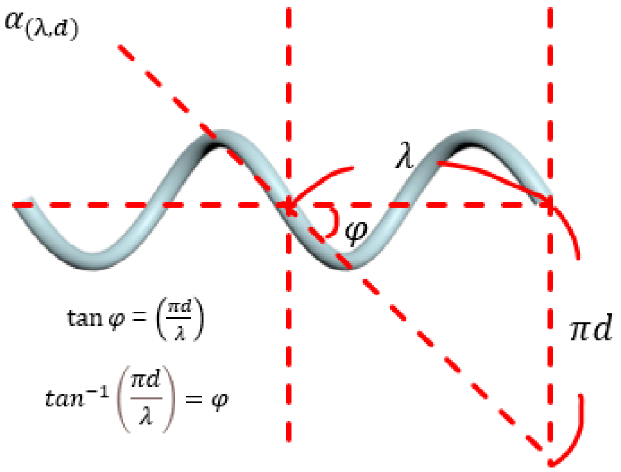

Figure S12. Illustration of a single HF where the helical angle (φ) can be calculated through trigonometric relationships.

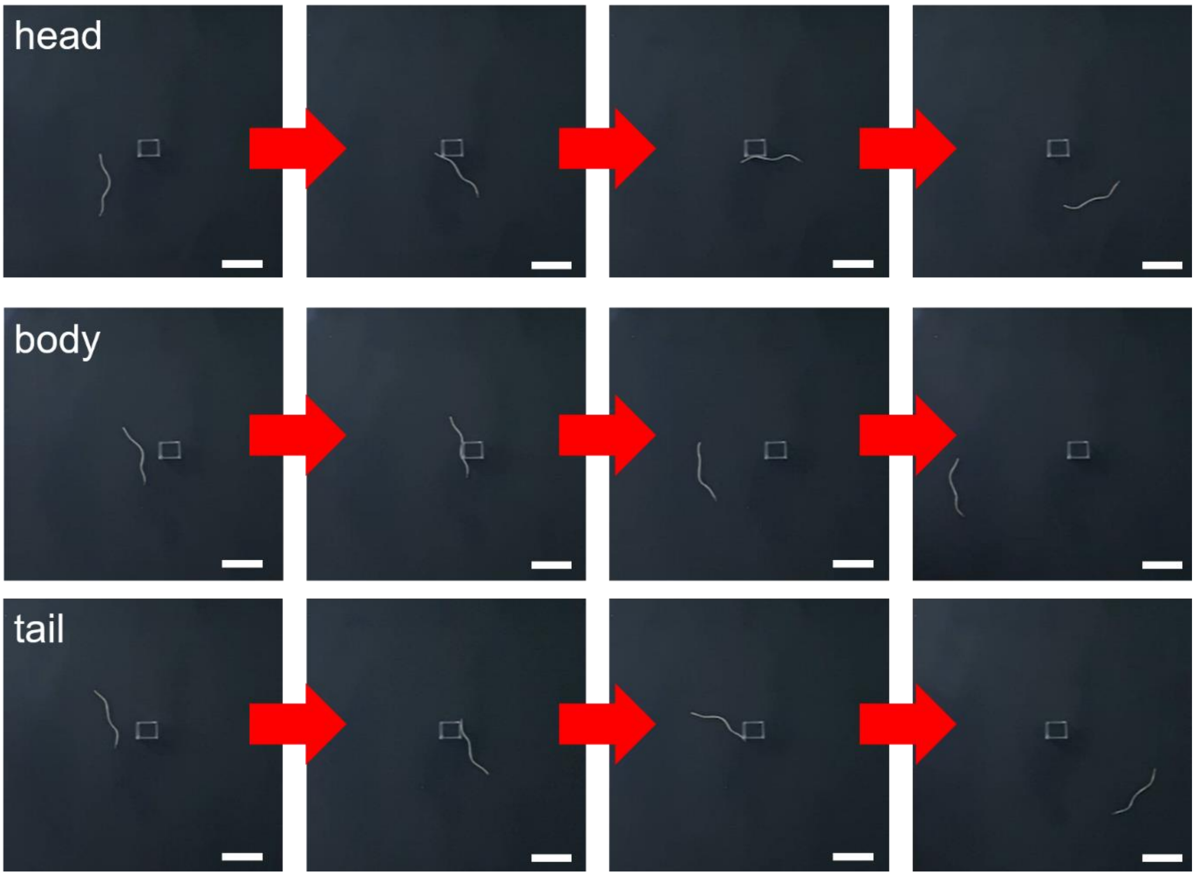

Figure S13. Photographs of the rolling locomotion of the LCE HF$_{\alpha(3,7)}$ in contact with a glass pillar at ~190 °C. Depending on how it encounters the pillar, LCE HF changes its path differently. Scale bars are 3 cm.

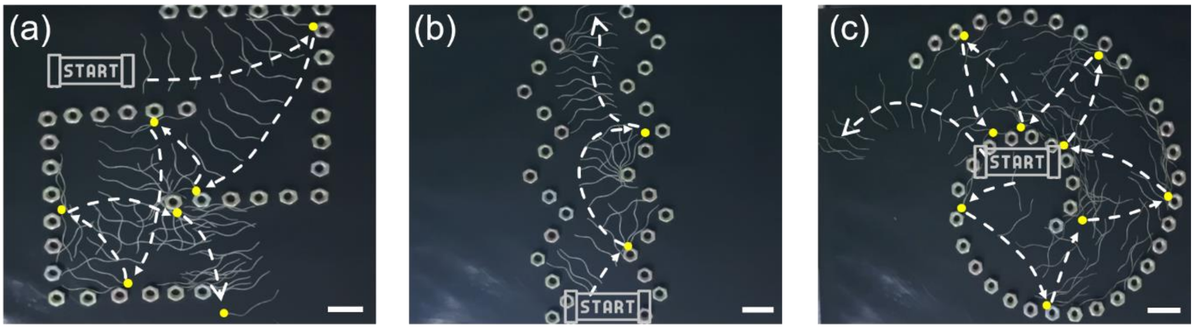

Figure S14. Optical images showing $HF_{\alpha(3,7)}$'s maze-escape ability with different obstacles on the hotplate ~190 °C: (a) zigzags, (b) vortexes, and (c) intersecting U-shaped terrains. Scale bars are 3 cm.

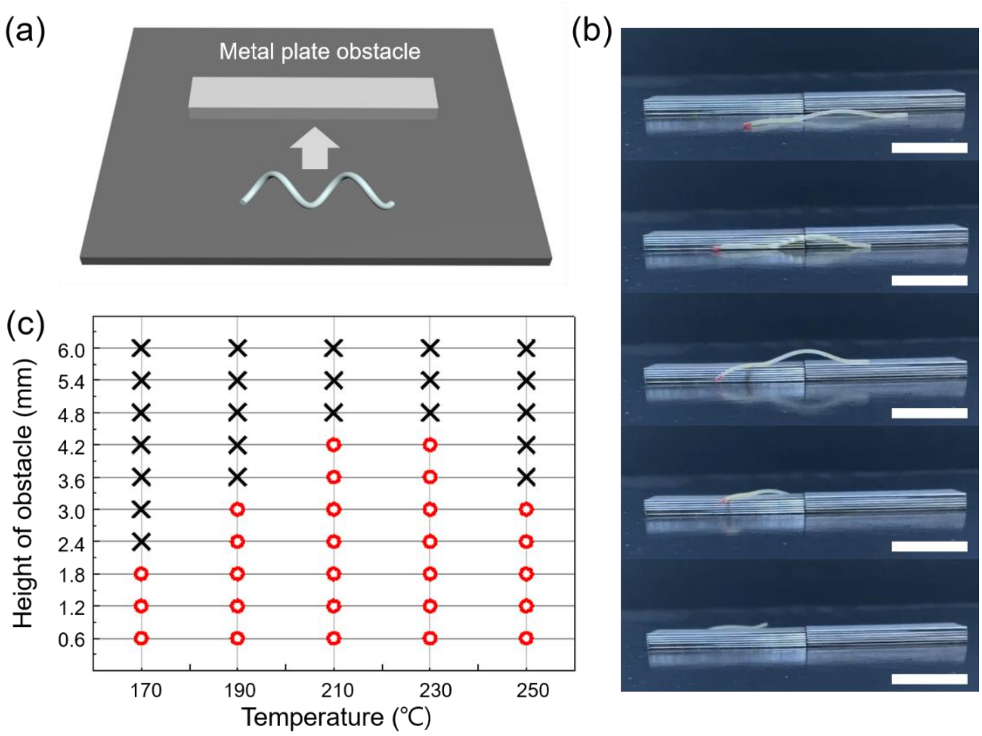

Figure S15. (a) Illustration and (b) photographs of $HF_{\alpha\,(3,\,7)}$ ascending a 3D staircase obstacle at 210 ℃. (c) The height profiles of the staircase that the HF overcomes. Scale bars are 3 cm.

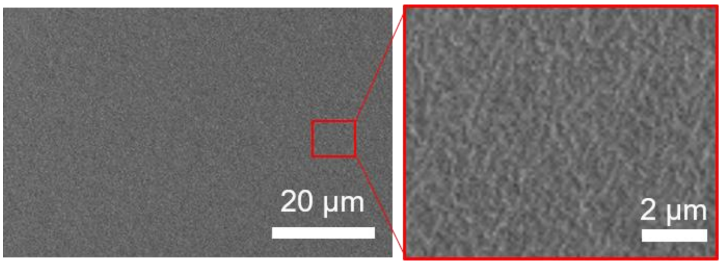

Figure S16. SEM images of PTFE coated fiberglass sheet.

Supporting Movie captions

Movie S1. The rolling locomotion of LCE HFs on a hotplate at 200 and 180 °C for $HF_{\alpha(3,7)}$ and $HF_{\beta(3,7)}$, respectively.

Movie S2. Locomotion of a toy model analogous to LCE HF rolling.

Movie S3. IR images following the rolling of LCE HF on a hotplate at 200 °C.

Movie S4. The self-sustainable rolling of LCE HFs for 1 hour at 200 °C.

Movie S5. Path changes of LCE $HF_{\alpha(3,7)}$ rolling around an obstacle at 190 °C.

Movie S6. Terrain exploration of LCE $HF_{\alpha(3,7)}$ at 190 °C.

Movie S7. Target finding of LCE $HF_{\alpha(3,7)}$ at 190 °C.

Movie S8. Maze escape of LCE $HF_{\alpha(3,7)}$ from various terrains at 190 °C.

Movie S9. LCE $HF_{\alpha(3,7)}$ ascending with stairs at 210 °C.
